# Child-caregivers’ body weight and habitual physical activity status is associated with overweight in kindergartners

**DOI:** 10.1186/1471-2458-14-822

**Published:** 2014-08-09

**Authors:** Sascha W Hoffmann, Suzan Tug, Perikles Simon

**Affiliations:** Department of Sports Medicine, Disease Prevention and Rehabilitation, Faculty of Social Science, Johannes Gutenberg-University Mainz, Media and Sport, Albert-Schweitzer-Str. 22, 55128 Mainz, Germany; Department of Sports Medicine/Sports Physiology, Institute of Sport Science, University of Bayreuth, Universitätsstraße 30, 95440 Bayreuth, Germany

**Keywords:** Child-caregivers, Association, Weight status, Habitual physical activity, Kindergarten teacher, Children’s weight status, Perception

## Abstract

**Background:**

The aim of this study was to examine whether child-caregivers’, both parents and kindergarten teachers, health parameters (age, weight status, habitual physical activity score) are significantly associated with the risk of overweight in young children.

**Methods:**

We assessed the individual body mass index standard deviation score in a regional cross-sectional health study and matched a representative sample of 434 kindergartners aged 3 to 6-years with their caregivers’ weight and habitual physical activity status. Furthermore, we identified factors associated with the general ability of child-caregivers to identify overweight in children, and the awareness to classify a child within the correct weight category.

**Results:**

Our study confirmed most of the known associations between parental anthropometrics and psychosocial factors with childhood overweight and obesity. A significantly higher proportion of boys tended to be overweight or obese (p = 0.027) and parents were more likely to misclassified boys overweight as normal weight (OR: 1.86; 95% CI 1.21-2.86). Adjusted for confounders, logistic regression analysis revealed that kindergarten teachers’ weight status (OR: 1.97; 95%-CI: 1.01-3.83) and habitual physical activity scores (OR: 2.32; 95%-CI: 1.10-4.92) were associated with children’s weight status.

**Conclusions:**

Kindergarten teachers’ weight and habitual physical activity score seem to be new independent risk factors for overweight in kindergartners 3 to 6-years of age. Our results suggest that the psychosocial, non-genetic association of non-parental child-caregivers on children’s weight is relatively high and that the association of non-parental child-caregivers warrants further investigation.

## Background

Prevalence of overweight and obesity in children has reached alarming figures with a worldwide estimate of 6.7% (95% CI: 5.6-7.7%) in 2010 and an expected estimate of 9.1% (95% CI: 7.3-10.9%) for 2020, which would correspond to about 60 million children [1]. In kindergartners Kalies et al. [2] reported that the overall prevalence of overweight (8,5%) and obesity (1,8%) in 1982 increased to 12.3% (overweight) and 2.8% (obese) in 1997. Latest national representative data from Germany revealed prevalence rates for overweight (obesity) of 9.5% (2.8%) in children between 2 and 6 years of age [3].

Although overweight in adulthood is significantly associated with the development of cardiovascular diseases [4], type 2 diabetes [5], and cancer [6], some studies revealed that being overweight is associated with a significantly decreased mortality [7]. However, becoming overweight as a child is a risk factor for obesity in adulthood, and obesity has been longitudinally related with higher mortality rates [8]. Moreover, cardiovascular risk factors are already present in overweight children, and obesity in childhood is associated with the development of coronary heart disease later in adulthood [9]. Thus, monitoring overweight and obesity in childhood, determining its risk factors and implementing tailored prevention-interventions is of high relevance for public health care [10–12].

Previous research has described early life determinants of overweight and obesity, such as maternal diabetes, maternal smoking (during pregnancy), parental overweight, rapid infant growth, no or short breastfeeding, no or short sleep duration, less than 30 min of daily physical activity, and consumption of sugar-sweetened beverages [12, 13]. One of the prevailing discussions about parental factors associated with overweight in children relates to the relative importance of the contribution of genetic or psychosocial factors [12, 14]. For example, parental overweight bears genetic and psychosocial dimensions that may explain the well documented association with overweight in children [15, 16]. For the development of prevention programs it is of high relevance to gain more knowledge about and to clearly demonstrate the importance of particularly the psychosocial dimension. A documented strong and robust association of the psychosocial dimension independent from the genetic dimension would principally justify a concentrated allocation of resources into early life overweight prevention. Unfortunately, psychosocial and genetic factors are well known to interact with each other in complex ways [17]. Therefore it is unlikely that one can succeed to reveal the relative importance of the psychosocial dimension by merely focusing the investigations on the interaction of children with their parents. Against the background that children spend a large part of their days at kindergartens, the time children attended the kindergarten together with the prevailing conditions in the kindergarten environment should come into the focus of public health research, which would include the prevention of overweight and obesity in kindergartens aged 3 to 6-years of age [18]. Against this background, recent research from *Hoffmann et al.* provide insights that kindergarten teachers have unfavorable health risk behaviors (e.g., decreased physical activity level, high screen time activities) and an increased risk for obesity [18]. Because kindergarten teacher’s health behaviors likely have a lasting effect on general health behaviors and future lives of their kindergartners, we hypothesized that kindergarten teachers’ weight and habitual physical activity status are significantly and independently from parental risk factors associated with children’s weight status. Here we have therefore assessed kindergarten teachers’ weight and habitual physical activity status along with the typical factors that are already known to be associated with childhood overweight in a representative kindergarten setting in a large German city with its typical city districts.

## Methods

### Study design

The Children Health Study of Mainz (CHSM) is a large, regional cross-sectional health study of kindergartners and their parents. The CHSM collected data of 869 children aged 2-7 years from 34 public kindergartens of the city Mainz, Germany. The CHSM aims to conduct additional information about health determinants associated with overweight and obesity in kindergartners and the anthropometrics and psychosocial factors of related caregivers. We differentiated “caregivers” in natural parents on the one hand and children’s kindergarten teachers in the respective kindergarten on the other hand. The CHSM investigated furthermore the association between anthropometric characteristics and fundamental movement skills (data not shown).

The data collection took place between May and July 2010 at the end of children’s individual kindergarten year. All children and their parents lived in one of the city districts of Mainz (≈200.000 residents). Similar to the Kindergarten Teacher Health Study (KTHS) [18], we used an active recruitment strategy to generate a high response rate. Parents from 34 public kindergartens in Mainz were informed and invited to participate in the study. Of the 869 parents we contacted, 558 replied to our invitation and completed a questionnaire. Six children were excluded because children lived with their grandparents or foster parents or adoptive parents. A further 118 were excluded due to incomplete information about body height and weight as well as about their habitual physical activity scores and individual socio-economic position. Thus, complete data became available for 434 parent-child pairs.

The questionnaires were filled in by one of the child primary guardians to perform informative meetings about the aims and procedures of the study in each kindergarten. Participation in this study was voluntary, and signed written informed consent was obtained from one parent or guardian of every participating child. We first obtained permission to perform the study from the supreme authority; the department head of social issues, children, youth, school, and health of the city of Mainz. The study then was approved by the Institutional Review Board (IRB) of the city of Mainz, which is responsible for evaluating the task of epidemiological research with personal data from participating subjects under ethical and legal aspects. Finally, the study was approved by the data protection commissioner according to the State Data Protection Act of Rhineland Palatinate, which is required for study performance.

### Questionnaire

One of the child parents reported to a self-constructed standardized paper and pencil questionnaire. Questions related to anthropometrics addressed child’s age, sex, language which is primarily spoken by the child, and height and weight at birth. Due to the fact that infants weight at birth is associated with later health outcomes, especially the risk for the development of obesity [19], children’s weight status at birth were classified into classifications defined by the Center for Disease Control and Prevention (CDC) [20]: very low birth weight (<1500 g), low birth weight (<2500 g), normal birth weight (<4000 g), and high birth weight (>4000 g). Additionally, children were classified into born small for gestational age (SGA), appropriate for gestational age (AGA), and large for gestational age (LGA) according to international proposed definition of -2SD (SGA), ±2SD (AGA), and +2SD (LGA) from the mean [21].

Questions related to psychosocial factors addressed potential determinants of overweight and obesity: habitual physical activity and screen time activity (characterized with daily television (TV) time and using the personal computer (PC)/Internet on weekdays/weekends) [18, 22].

Children’s and child-caregivers’ habitual physical activity scores were assessed using a modified Baecke habitual physical activity questionnaire (HPAQ) [23] in which parents reported about their child’s and their own physical activity behavior. There were some small modifications in the formulations of some items, especially regarding the work index [24]. The work index was replaced by a “kindergarten index”, similar to the examination of *Vogels et al.* [25]. Although the HPAQ has been used in previous research in different samples of children and adolescents [24–26], this method of evaluation of physical activity in kindergartners with the HPAQ has been applied for the first time. However, the HPAQ was previously validated for the assessment of physical activity [27, 28]. Questions about screen time activity have already been used in questionnaires within the German Health Interview and Examination Survey for Children and Adolescents (KiGGS, 2003-2006) [22, 29]. Information about children’s screen time activities were categorized into three groups as follows: children’s TV/PC time less than 1 h/day, TV/PC time between 1-2 h/day and TV/PC time 2 h/day or more whereas parental screen time activities were divided into two groups: parents with TV/PC time less or equal than 2 h/day and those with more than 2 h daily.

One of the child primary guardians reported furthermore to questions retrieving information about both, the primary responder to the questionnaire, and their respective spouse. In particular, these were questions about anthropometric characteristics (age, sex, weight, height), cultural background (country of birth, nationality), smoking status, and educational level.

Questions about screen time activity behavior, habitual physical activity, daily parental child-care behavior, eating behavior patterns, and parental perception of weight status and health risks associated with overweight and obesity in children were only addressed to the primary responder of the questionnaire.

Daily parental child-care behavior patterns were as follows: “time of active child care”, “reading to my child”, “talking with my child about things that it has experienced during the day”, “to inform about what my own child has eaten during the day”, “my child is allowed to watch TV, if it is boring” (1 = never, 2 = seldom, 3 = sometimes, 4 = often, 5 = always). The different eating behavior patterns in daily life were as follows: “eating together with the family”, “eating breakfast”, and “watching TV during meals” (1 = never, 2 = seldom, 3 = sometimes, 4 = often, 5 = always). All these variables were introduced as categorical variables (never/seldom vs. sometimes/often/always) in the analyses. In the style of the study from *Rapp et al.* [30], participants responded also when they typically pick up their child from kindergarten (type of kindergarten) to calculate the daily time children attended the kindergarten.

In addition, information about health related issues during pregnancy (gestational weight gain, smoking status, alcohol intake) and breastfeeding (smoking, duration of exclusive breast feeding) were assessed.

Parental BMI status was calculated according to the WHO criteria [31]. Educational level was used as a proxy for the individual socio-economic position (SEP), and was categorized into three levels: low educational level (no education, to do an apprenticeship or secondary modern school), middle educational level (vocational- and technical school or secondary modern school), high educational level (University degree or technical college degree) as described previously [32].

Questions of parents perception of children’s weight status and its association with physical and mental health risks were taken from the study of *Warschburger and Kröller* [33], and assessed in the same way as recently published in the Kindergarten Teacher Health Study (KTHS) [18]. Therefore, parents were presented a panel of 7 silhouettes to rate their perception of children’s weight status. According to proposed age and sex-specific cut-offs, two silhouettes represented underweight children (3rd and 10th percentiles), three silhouettes represented children within the normal-weight range (25th, 50th, and 75th percentiles), and two silhouettes represented an overweight and obese child (90th and 97th percentiles) [33]. Parents were required to answer the following questions: “Which of the silhouettes do you think represents an overweight child?”, and, in addition, they were questioned about their perception of the physical or mental health problems associated with being overweight (e.g., “Which silhouettes do you think have an increased risk for physical/mental health problems?”). Silhouettes above the 90th percentile were defined as overweight and represented a higher health risk. Furthermore, parents had to reply to one more question: “Which silhouette best represents the weight status of your child?”

Finally, parents were asked two more questions about the perception of weight status given from external people: “Did anybody tell you that your child is overweight?” and “Did anybody tell you that you are overweight?” As given answers we presented: “Physician”, “relatives” and “friends”, and “other people”.

### Assessment of anthropometric measurements in children and definition of overweight and obesity

Children were weighed by professional trained staff. Height and weight was measured with a calibrated portable stadiometer (accurate to 0,5 cm) (SECA 217 [SECA, Hamburg, Germany]) and a calibrated flat scale (accurate to 100 g) (SECA 803). Children were weighed without shoes and wore light clothing. *Monasta et al.* suggested that the IOTF-reference could be more preferable for the identification of overweight and obesity in pre-school children [34]. Thus, BMI was calculated and overweight and obesity were conducted according to the IOTF international proposed age- and sex-specific cut-offs [35]. Height, weight, BMI, and WC standard deviation scores (SDS) were conducted according to the internationally common used UK 1990 reference [36–38].

### Social environment of the kindergarten

Taking the inner-city social environment into account, we tested the hypothesis that kindergartners who attended kindergartens which are located in deprived city districts were more likely to be overweight or obese. As part of a social area analysis of the city of Mainz conducted by *Pfeiffer et al.* [39] comprehensive information about general conditions (land use, living situations, demography, budgetary structure and family, employment and income maintenance, and highly problematic groups and education) and social structures were collected from each of the 65 city districts. Based on these data, the life situation index (LSI) was created to combine all relevant characteristics into one specific scale [39]. The LSI consists of 4 thematic indices (employment/working life, education, social situation/heterogeneity, and home environment) that are weighted differently. The LSI may be an effective tool for demonstrating socio-economic differences within specific geographic areas of the city of Mainz. Detailed description and method of calculation of the LSI is presented also in the Kindergarten Teacher Health Study [18].

### Statistical analysis

Descriptive characteristics of total available parental reports and the final parental sample were employed and stratified by sex. Children’s anthropometric characteristics were calculated for the total sample, and were presented as means (SD) and relative frequencies. Sex differences were analyzed with the independent t-test, and for not normally distributed variables with the nonparametric Mann-Whitney-U-Test. For categorical data, sex differences were tested with the Chi-square test. Kruskal-Wallis-ANOVA and oneway ANOVA with Bonferroni post hoc test were carried out for the comparison between children’s anthropometric characteristics.

Factors associated with overweight in kindergartners were examined using a multiple logistic regression analysis with odds ratios (ORs) and the corresponding 95% confidence intervals (CI). The selection of covariates was based on the literature for children’s overweight status and on their availability in the sample data [12, 40]. Continuous variables were categorized. Variables of high interest which possibly affect children’s weight status were age, overweight status, and habitual physical activity scores of children’s kindergarten teachers from the respective public kindergartens. Kindergarten teachers mean ± SD scores of each kindergarten were categorized and matched with the individual BMI SDS of each child and then entered simultaneously in the regression model.

Statistical analysis of parents’ perception of children’s weight status were similarly employed according to *Warschburger and Kröller* [33]: Chi-square tests were used to examine significant group differences in the frequency of the presented silhouettes, and logistic regression analysis were conducted to provide odds ratios (95% CI) for the estimated variables and to predict determinants of parents who were not able to correctly identify overweight in general and parents who mismatched their own child’s weight status. JMP 8.0 (SAS, Cary, NC) and PASW Statistics 18.0 (formerly SPSS, Chicago, IL) were used for further statistical data analyses, and overall power analysis was sufficient and indicated a power ranged from 97-99% with an alpha of 0.05.

## Results

Table 1 presents the descriptive characteristics of the study sample, separately for boys and girls. About 56.0% of the children were boys and mean age was 4.9 ± 1.0. Girls were significantly older (p = 0.009), whereas BMI were higher in boys (p = 0.043). As excepted, prevalence of overweight and obesity was significantly higher in boys than in girls (p = 0.027). The overall prevalence of overweight in children according to the IOTF cutoffs was 18.1%, whereas 4.4% of them were obese, respectively. Furthermore, 5.5% of the children were underweight, 76.5% were of normal weight, and height, weight and BMI increased gradually over the whole age period (data not shown). The two samples of children with (n = 434) and without (n = 311) information from the parent rated questionnaire differed only significantly from each other concerning the mean age in years (4.9 ± 1.0 vs. 5.1 ± 1.0, p = 0.026). No differences were found regarding BMI status (p = 0.271).

**Table 1:** **Descriptive characteristics (Mean ± SD** ^**a**^ **) of the total children study sample stratified by sex**

Item	Boys	Girls	P*	Total
N	243	191		434
Age	4.8 ± 1.0	5.0 ± 1.0	**0.009** ^**†**^	4.9 ± 1.0
Height, cm	109.8 ± 7.8	110.5 ± 8.5	0.349^‡^	110.1 ± 8.3
Height SDS	0.5 ± 1.1	0.4 ± 1.0	0.641^‡^	0.4 ± 1.0
Weight, kg	19.6 ± 3.8	19.4 ± 3.5	0.910^†^	19.5 ± 3.7
Weight SDS	0.5 ± 1.1	0.3 ± 0.9	0.158^†^	0.4 ± 1.0
BMI, kg/m^2^	16.2 ± 1.6	15.8 ± 1.4	**0.043** ^**†**^	16.0 ± 1.5
BMI SDS	0.3 ± 1.2	0.1 ± 0.9	0.109^†^	0.2 ± 1.0
Thinness grade I, % (n)^b^	4.9 (12)	6.3 (12)	0.027**	5.5 (24)
Normal weight, % (n)	72.8 (177)	81.2 (155)		76.5 (332)
Overweight, % (n)	15.6 (38)	11.0 (21)		13.7 (59)
Obese, % (n)	6.6 (16)	1.6 (3)		4.4 (19)

^a^SD = Standard deviation.

^b^Thinness Grade I-III were combined into one single thinness grade I due to small sample size.

*P < 0.05. Values in bold indicate statistical significance.

^†^Mann-Whitney-U-Test.

^‡^Independent t-test.

**χ^2^-test.

Additionally, significant mean differences of anthropometrics between BMI groups were observed in both boys and girls, respectively (data not shown).

The anthropometric sex differences were also observed in the excluded children sample. Almost 30% of the overweight and obese children had parents classified into the low educational level group, while nearly 45% of obese children were classified into the high educational level group (P for difference <0.001).

Table 2 shows the total available parental characteristics of age, body height, body weight, calculated BMI groups, smoking status, country of birth, nationality, and educational level from the questionnaire stratified by sex. The related data of children’s kindergarten teachers were taken from results of the Kindergarten Teacher Health Study (KTHS) [18]. Mean ± SD age was 38 ± 6.0 years for the parents and 37.2 ± 10.6 years for the kindergarten teachers. Mothers and fathers differed significantly regarding body height, body weight and BMI. Prevalence of overweight (obesity) was 52.5% (8.5%) for fathers, 33.3% (11.4%) for mothers, and 41.2% (17.9%) for kindergarten teachers. About 26% of fathers and 20% of mothers were current smokers with an average consume of 13 and 8 cigarettes daily, respectively. Furthermore, about 15% of the parents were former smokers. Data of the KTHS show that about 26.8% of kindergarten teachers were current smoker with an average consume of 10 cigarettes daily, whereas 19.2% were former smokers. Twenty-seven percent of the parents were born abroad and almost 20% were of non-German nationality, while 15.3% of kindergarten teachers were born abroad. Classifications of SEP indicated that nearly 30% of the parents were classified as low educational level, while about 55% were classified as high educational level.

**Table 2:** **Descriptive characteristics of total available parental reports stratified by sex (n = 830) compared to data of the KTHS (n = 313)** ^**a**^

Item	Mothers				Fathers				Kindergarten teachers			
	n	%	Mean	SD^b^	n	%	Mean	SD^b^	n	%	Mean	SD^b^
Age, years*	432	99.5	36.3	5.4	398	91.7	39.7	6.5	313	100.0	37.2	10.6
Height, cm*	428	98.6	167.8	6.3	388	89.4	179.9	9.5	313	100.0	167.1	7.2
Weight, kg*	420	96.8	67.4	13.0	379	87.3	83.4	12.6	313	100.0	70.8	15.8
**BMI, kg/m** ^**2**^ *	420	96.8	24.1	4.5	379	87.3	25.6	3.2	313	100.0	25.4	5.5
**BMI** ^**b**^ **groups** ^**c**^ *												
Underweight	17	4.0	-	-	3	0.8	-	-	10	3.2	-	-
Normal weight	263	62.6	-	-	176	46.7	-	-	174	55.6	-	-
Overweight	92	21.9	-	-	166	44.0	-	-	73	23.3	-	-
Obese	48	11.4	-	-	32	8.5	-	-	56	17.9	-	-
**Smoking status** ^**d**^												
Current smoker*	88	20.4	-	-	106	26.1	-	-	84	26.8	-	-
Number of cigarettes/day*	84	95.5	8.0	4.9	99	93.4	13.1	9.0	80	95.2	10.4	6.0
Nonsmoker*	344	79.6	-	-	300	73.9	-	-	229	73.2	-	-
Former smoker/Stop smoking (yrs)	48	14.0	7.5	4.6	49	16.3	8.0	6.4	60	19.2	8.0	5.9
**Country of birth**												
Germany	315	72.7	-	-	306	72.5	-	-	265	84.7	-	-
Foreign country	118	27.3	-	-	116	27.5	-	-	48	15.3	-	-
Nationality*												
German	340	78.5	-	-	345	81.6	-	-	-	-	-	-
Non-German	93	21.5	-	-	78	18.4	-	-	-	-	-	-
**Socio-economic position** ^**e**^ *												
Low educational level	138	31.8	-	-	112	27.4	-	-	-	-	-	-
Middle educational level	64	14.7	-	-	60	14.7	-	-	-	-	-	-
High educational level	232	53.5	-	-	237	57.9	-	-	-	-	-	-

^a^Modified data of kindergarten teachers are taken from the Kindergarten Teacher Health Study (KTHS) by Hoffmann et al. [18].

^a^SD = Standard deviation.

^b^BMI = Body mass index.

^c^BMI groups according to WHO [31].

^d^Classification according to DESTATIS [41].

^e^Socio-economic position is classified according to Sacerdote [32].

*Sex differences significant at p ≤ 0.05.

Primary responder to the questionnaire were mostly mothers and they responded to personal questions about items of habitual physical activity, screen time activity, overweight status, and about the perception of children’s weight status and its associated health risks. Results and comparable data of the KTHS [18] are shown in Table 3. The majority of parents analyzed in the final sample (n = 434) were female (85.7%). Indices of habitual physical activity and the general perception of weight status and associated health risks did not differ between sexes. Prevalence of overweight was more frequent in fathers compared to corresponding mothers (53.3% to 35.4%; p = 0.008). Moreover, fathers had higher frequencies of PC/Internet use on weekends than mothers (p = 0.007). Results of the KTHS indicated that about 40% of German kindergarten teachers were overweight or obese. Additionally, mean ± SD leisure time habitual physical activity scores were lower in German kindergarten teachers compared to the parental sample, and in addition, “[…] kindergarten teachers were more likely to participate in more screen time activities on weekends than on weekdays” [18].

**Table 3:** **Final parental sample descriptions of the items of HPA** ^**a**^ **, screen time activity, overweight status, and parental perception of weight status and associated health risks stratified by sex (n = 434)**

Item	Mothers				Fathers				P*	Kindergarten teachers^b^			
	n	%	Mean	SD^c^	n	%	Mean	SD^c^		n	%	Mean	SD^c^
**Habitual physical activity scores** ^**d,e**^	371	99.7			62	100				193	61.7		
Work	-	-	2.6	0.7	-	-	2.6	0.8	0.825	-	-	2.8	0.4
Sport	-	-	2.6	0.7	-	-	2.7	0.5	0.196	-	-	2.7	0.6
Leisure-time	-	-	3.2	0.6	-	-	3.1	0.6	0.236	-	-	2.7	0.7
HPA score	-	-	8.3	1.4	-	-	8.4	1.2	0.606	-	-	8.3	1.2
**Screen time activity** ^**f**^	372	100.0			62	100.0							
TV/DVD on weekdays, ≤2 h/daily	334	89.8	-	-	55	88.7	-	-	0.822	232	76.1	-	-
TV/DVD on weekdays, >2 h/daily	38	10.2	-	-	7	11.3	-	-		73	23.9	-	-
TV/DVD on weekends, ≤2 h/daily	287	77.2	-	-	45	72.6	-	-	0.423	150	49.5	-	-
TV/DVD on weekends, >2 h/daily	85	22.8	-	-	17	27.4	-	-		153	50.5	-	-
PC/Internet on weekdays, ≤2 h/daily	296	79.6	-	-	43	69.4	-	-	0.096	284	92.5	-	-
PC/Internet on weekdays, >2 h/daily	76	20.4	-	-	19	30.6	-	-		23	7.5	-	-
PC/Internet on weekends, ≤2 h/daily	339	91.1	-	-	49	79.0	-	-	**0.007**	255	83.9	-	-
PC/Internet on weekends, >2 h/daily	33	8.9	-	-	13	21.0	-	-		49	16.1	-	-
**BMI status** ^**f**^	362	97.3	-	-	60	96.8	-	-					
Overweight and obese	128	35.4	-	-	32	53.3	-	-	**0.008**	129	41.2	-	-
**Perception of weight status and associated health risks** ^**f**^	371	99.7			62	100.0				312	99.7		
General ability to identify overweight silhouettes	172	46.4	-	-	28	45.2	-	-	0.861	-	44.6	-	-
Parents and kindergarten teachers who identified overweight silhouettes as at risk for physical health problems	125	33.7	-	-	20	32.3	-	-	0.825	-	40.1	-	-
Parents and kindergarten teachers who identified overweight silhouettes as at risk for mental health problems	88	23.7	-	-	9	14.5	-	-	0.108	-	21.2	-	-

^a^HPA = Habitual physical activity according to Baecke et al. [23].

^b^Modified data of health risk behaviors of kindergarten teachers are taken from the Kindergarten Teacher Health Study (KTHS) by Hoffmann et al. [18].

^c^SD = Standard deviation.

^d^Sex differences tested with the Mann-Whitney-U-Test.

^e^Sex differences tested with the independent t-test.

^f^Sex differences tested with the χ^2^-test.

*P < 0.05. Values in bold indicate statistical significance.

Table 4 demonstrates the results of the regression analysis providing factors associated with overweight in kindergartners. Main results indicate that kindergarten teachers’ BMI status and habitual physical activity score significantly contributed to childhood overweight at pre-school age. Moreover, the risk of being overweight at pre-school age increases about 2.32-fold (95% CI: 1.10-4.92, p = 0.028) if children’s kindergarten teachers have low habitual physical activity scores. The time children spend per day in the kindergarten is significantly correlated with the prevalence of overweight in children 3 to 6-years of age (OR: 0.05; 95% CI: 0.01-0.52, p = 0.012). Additional variables who did show a significant positive associations with childhood overweight included being a boy, parental BMI and low parental habitual physical activity scores, maternal smoking during pregnancy, television use during meals as well as children’s TV/DVD time on weekends, and parental PC/Internet use on weekdays and on weekends.

**Table 4:** **Factors associated with overweight in kindergartners aged 3 to 6-years** ^**a**^

Variables	OR^b^	95% CI for OR^c^	P*
**Kindergarten teachers BMI status** ^**d**^			
Overweight	1.97	1.01 - 3.83	0.047
Normal weight	1.00		
**Kindergarten teachers Habitual Physical Activity (HPA) score** ^**d,e**^			
6.83-8.23	2.32	1.10 - 4.92	0.028
8.30-9.94	1.00		
**Sex**			
Boy	2.53	1.17 - 5.50	0.019
Girl	1.00		
**Maternal BMI**	1.10	1.01 - 1.20	0.033
**Paternal BMI**	1.29	1.15 - 1.45	<0.001
**Parental Habitual Physical Activity (HPA) score** ^**d**^			
4.88-8.25	2.54	1.02 - 6.35	0.045
8.38-14.38	1.00		
**Maternal smoking during pregnancy**			
No	0.05	0.03 - 0.94	0.045
Yes	1.00		
**Children’s TV/DVD time (on weekends)**			
<1 h daily	0.11	0.02 – 0.73	0.022
1-2 h daily	0.13	0.02 – 0.73	0.020
>2 h daily	1.00		
**Parental PC/Internet time (on weekdays)**			
≤2 h daily	0.27	0.09 - 0.89	0.030
>2 h daily	1.00		
**Parental PC/Internet time (on weekends)**			
≤2 h daily	0.10	0.02 - 0.72	0.022
>2 h daily	1.00		
**Television use during meals**			
Never/seldom	0.36	0.13 - 0.98	0.035
Sometimes/often/always	1.00		
**Daily time children attended the kindergarten**			
until 12:00 (noon)	0.47	0.08 - 2.56	0.381
until 2:00 pm	0.05	0.01 - 0.52	0.012
until 4:00 pm	0.44	0.09 - 2.11	0.301
around 4:30 pm	0.38	0.08 - 1.89	0.237
5:00 pm and later	1.00		

^a^Only significant associations are displayed.

^b^OR = Odds ratios.

^c^OR and 95% confidence intervals (CI) are from multiple logistic regression analysis in which all independent variables were included simultaneously: Kindergarten teachers BMI, kindergarten teachers age, kindergarten teachers habitual physical activity, kindergarten teachers habitual slothful behavior index, children’s sex, children’s migration background, educational level, parental BMI, parental habitual physical activity, children’s total fundamental movement skill score, habitual slothful behavior factors 1-3, smoking during pregnancy, duration of exclusively breastfeeding, birth weight, born small for gestational age, social environment of the kindergarten (LSI), children’s screen time activities, daily parental child-care behavior, parental screen time activity behavior, daily time children attended to the kindergarten.

^d^Data assessed in the KTHS (Hoffmann et al. [18]).

^e^Habitual physical activity score was calculated by the work index, the leisure-time index, and the sport index. Scores were dichotomized. The lower the score, the more likely the participant had lower physical activity levels.

*Significant difference at p ≤ 0.05.

Silhouettes associated with overweight in children were correctly recognized and categorized by 45.8% of the respondent parents, and 44.6% of the kindergarten teachers (Table 3). Demographic variables like children’s age, children’s and parental weight status, educational level and migration background were not significantly associated with parents’ ability to identify overweight silhouettes correctly.

If parents were asked to classify their own child with the matched silhouettes, 52.1% identified their own child within the right weight category. However, only 16.7% of the parents of an obese child were able to identify their own child as obese, and strikingly, in only 5.3% of the cases parents of overweight children correctly identified their own child as overweight, compared to parents of normal weight children who had in 62.8% of the cases the general ability to indicate their own child correctly with the matched silhouettes. Furthermore, 79.1% of the parents of underweight children were able to estimate the correct silhouettes. Results of the forward stepwise logistic regression analysis indicate that parents of boys, overweight children, and parents who did not identify correctly silhouettes of physical health risks associated with overweight were significantly less able to correctly identify overweight in general, and were significantly less able to identify their own child’s weight status (Table 5).

**Table 5:** **Logistic regression model** ^**a**^ **of parents who are not able to correctly identify overweight in general and parents who did not matched their child’s silhouette correctly**

Variables	OR^b^	95% CI for OR^c^	P*
**Step 1: Sex**			
Boy	1.86	1.21 - 2.86	0.047
Girl	1.00		
**Step 2: Weight status**			
Own child is overweight	1.79	1.02 – 3.12	0.040
Own child is of normal weight	1.00		
**Step 3: Associated health risks**			
Incorrect identification of physical health risks associated with overweight	1.75	1.06 - 2.88	0.027
Correct identification of physical health risks associated with overweight	1.00		

^a^Results of a forward stepwise logistic regression model.

^b^OR = Odds ratio.

^c^OR and 95% confidence intervals (CI) are from forward stepwise regression analyses in which all independent variables listed were included in the model simultaneously.

*Significance at p < 0.05.

Furthermore, parents had to answer questions which are related to the awareness in identifying overweight in children by external family-related persons (physician, relatives, friends, and others). If parents have an overweight child, only about 20% of family-related people told the parents that their child is overweight. Of these, in about 50% of the cases the physician informed the parents of the child being overweight, followed by relatives (18.8%), and other persons (12.5%). 18.8% of the respondents reported that all persons named above told them that their own child is overweight.

## Discussion

The aim of this study was to specify the role of children’s caregivers, both their parents and their kindergarten teachers, as mediating factors associated with overweight and obesity in kindergartners aged 3 to 6-years. Interestingly, regression analysis indicated that children’s kindergarten teachers’ weight status and habitual physical activity score significantly contribute to children’s overweight status. The risk of being overweight in kindergartners was about 1.97 times (95% CI: 1.01-3.83, p = 0.047) and 2.32 times (95% CI: 1.10-4.92, p = 0.028) higher if their kindergarten teachers are overweight themselves or have low physical activity scores, respectively. Up to now, studies which investigated the well-known factors associated with overweight in children primarily focused on genetic aspects [42, 43] as well as children’s home and neighborhood environment [44, 45].

Since children spent a large part of their day in child-care centers, current studies should additionally focus on the association of the child-care center environment on children’s health. In a recently published paper and from a new point of view, we initially reported about high obesity prevalence and unfavorable health risk behaviors of German kindergarten teachers [16]. Main factors associated with kindergarten teachers’ obesity status were lack of physical activity, the presence of high screen time activities, especially on weekends, and working in socially deprived city districts. These results required further attention for the implementation of future child health prevention strategies in general, because “[…] kindergarten teachers have a particularly high interaction with the general public, and due to their daily contact with children, kindergarten teachers’ health behaviors likely have a lasting effect on the general health behaviors and future lives of their kindergartners; […] hence, time spent in kindergarten and primary school may be important for the timing of future public health approaches” [18].

Kindergarten teachers nowadays play a key role in health promotion, and therefore, it is important to implement interventions that promote physical activity and general health patterns in very young children attended regularly to the kindergarten. This issue is complex and of high interest, because kindergarten teacher themselves have below average physically activity levels, and have high screen time activities, especially on weekends [18]. Moreover, kindergarten teachers’ health behaviors seem to be significantly associated with children’s weight status as we show here for the first time. It is tempting to speculate that kindergarten teachers that are overweight and have low habitual physical activity scores probably promote overweight in early childhood. However, we need to point out that our study is not able to confirm or reject any causal relationship between these factors of the kindergarten teachers and the weight status of the respective kindergartners. There might be indirect and unknown factors that may contribute to aforementioned association. Such typical confounding factors might be the social status, linked with the city district the kindergarten is situated, and linked with the overweight of the kindergarten teacher. We were not able to confirm such an indirect relationship in our study, since the aforementioned association was robust against adjustment for these potential confounders.

Present knowledge about the association kindergarten teachers may have on children’s weight status is still limited. Nevertheless, our study results strongly suggest focusing on this aspect more precisely in well-controlled longitudinal studies of adequate sample size. For the time being, it seems to be advisable that politicians and health care decision makers start thinking about intensifying not only theoretical but also practical education about weight control and physical education of kindergarten teachers [18].

As already reviewed by *Monasta et al.* [12], regression analysis indicated significant influencing factors associated with overweight in 3 to 6-year-old kindergartners as shown as follows: children’s sex, parental BMI, parental habitual physical activity score, maternal smoking during pregnancy, children’s and parental screen time activity, and TV use during meals.

Although there is some limitation regarding the relatively small sample size, the study outcomes are in good accordance with results which have previously been published: In agreement with *Kosti et al.*, parental BMI significantly contributed to child’s overweight status [46]. Children’s screen time activity was also positively associated to overweight status [30, 40, 47–49] as well as parental PC use both on weekdays and on weekends. *Kourlaba et al.* [47] furthermore suggested that parental TV viewing time is associated with children’s TV viewing time, and additionally, the current findings determined a relationship to overweight status in kindergartners. Moreover, TV use during meals also contributed significantly to overweight status in kindergartners. This is also in good accordance with results provided by *Dubois et al.*, who reported about a relationship between TV use during meals and overweight among kindergartners [40].

Furthermore, low parental habitual physical activity scores were significant predictors of childhood overweight (OR: 2.54, 95% CI: 1.02–6.35, p = 0.045). These findings are consistent with previous research conducted by *Taylor et al.,* who found also a positive association between parental activity and child’s activity (r = .17-.28) [50]. *Eriksson et al.* found similar associations of parent-child physical activity relationships between 12-year-olds and their parents [51]. Parental physical activity was also assessed with the HPAQ, while children’s physical activity was administered with general questions about sports participation and further vigorous activities. They conclude that the family environment is “[…] an important target for interventions to increase physical activity in children […]” [51]. In this connection, *Dunton et al.* [52] suggested that joint physical activity in parent-child pairs could have health benefits for both children and parents, “[…] especially for girls, older children, older parents, and higher income families”. Moreover, *Hnatiuk et al.* suggested that implementation of physical activity interventions has to start as early as possible, and predominantly focus on certain times of the day, particularly in the morning and in the afternoon until about 4:00 pm [53]. In addition *Van Cauwenberghe et al.* [54] proposed that “weekdays hold the greatest promise for improving […] sedentary behaviors and moderate-to-vigorous physical activity (MVPA)”. The morning hours till the early afternoon shall be deemed to be opportunities to promote MVPA in pre-school aged boys and girls. This time period is the period during which children attended the kindergarten. In this context and in contrast to the study of *Rapp et al.* [30], daily time children attended the kindergarten was positively associated with children’s overweight status. Children who attended the kindergarten until 2:00 pm had the lowest likelihood of being overweight compared to other time spans. Children who did not regularly attend the kindergarten in the afternoon may possibly reflect that they have higher physical activity levels due to the fact of attending afternoon play groups, special courses of sport clubs or have higher joint parent-child physical activity [52]. In contrary, *Finn et al.* [55] reported about factors associated with physical activity in pre-school children and pointed out that the child-care center was the strongest predictor of activity levels in children, with more than 50% of the average daily activity counts occurring between 9:00 am and 5:00 pm.

In our study, only 46.5% were able to identify the overweight silhouettes, and 33% (19.1%) identified the overweight silhouettes as at risk for physical (mental) health risks. Parents and respective kindergarten teachers of their children had the same ability to correctly identify overweight silhouettes. The current findings indicate that parents of boys, overweight children and parents who did not identify correctly silhouettes of physical health risks associated with overweight, were significantly less able to correctly identify overweight in general, and were significantly less able to identify their own child’s weight status. This issue seems to be problematic. It is tempting to speculate that not identifying overweight in young children, especially in young boys, could also be possibly involved within the complex system of associated factors responsible for the development of overweight and obesity later in adulthood. Latest national data from the “German Health Interview and Examination Survey for Adults” (DEGS1) indicated that being overweight is more prevalent in men than in women. According to DEGS1, 67.1% of men and 53.0% of women are overweight [56]. The results of our analysis may possibly support this hypothesis because only about 5% of parents from overweight children recognized their own child as overweight. In contrast, about 80% of parents from underweight children classified their own child as underweight. It seems to be that German parents were more sensitized for the prevention of underweight than overweight in children.

Additionally, parents from normal weight children more often underestimated their own child’s weight status than others did, in particular in boys. We propose that this may be an indicator for possible changes in the perception of the general public. Our results concerning the general ability of external family-related people to identify overweight children may contribute to this hypothesis; our findings show that only in 20% of the cases family-related people told the parents that their own child is overweight.

According to the IOTF cutoffs [35], 18.1% of the children were overweight, while 4.4% of them were obese. Although there is recent evidence that childhood overweight and obesity prevalence are plateauing or even declining in some countries [57], results of the present study show still high overweight prevalence rates in kindergartners. Data from nine countries were presented recently by *Olds et al.* [57], however, data for kindergartners were only available from France (12.1% and 3.1% of overweight and obesity prevalence of 5 to 6-year-olds in 2005), the Netherlands (10.9% of overweight prevalence in 3 to 6-year-old girls) and the US (about 16% of overweight prevalence in 2 to 5-year-olds). Besides a critical life span in children’s life for a possible increase in the prevalence of overweight in kindergartners, the determination of associated factors is of high importance for future public health approaches.

Besides, our study tested whether there are factors associated with overweight in kindergartners and whether children’s weight status is associated by their caregivers’ weight and habitual physical activity scores within a cross-sectional design and by quantitative research measurements. Interpretations should be drawn with caution: The findings of this study are subject to its cross-sectional design. Larger longitudinal observational studies are needed to draw causal relationships and quantify our results. Limitations of the study particularly include the use of self-reported height and weight of both parents and children’s kindergarten teachers [18]. Participants’ self-reported information from the questionnaire might lead to false conclusions being drawn, because people tend to underreport their body weight, especially the obese subjects [18, 58–60]. This leads to underestimates of overweight and obesity prevalence, and results may be biased.

The strength of the present study is the high numbers of participating individuals, especially the large number of children with direct measurements of body height and weight, from a representative local area as well as the high number of confounding variables used in our statistical analysis. Although study participation rates have declined during the last decades, the response rate is similar to response rates reported in the epidemiological literature [61]. Our study provides evidence that besides the already well known genetic and environmental factors associated with overweight and obesity in children, children’s kindergarten teachers’ weight status and habitual physical activity scores significantly correlate with overweight status in kindergartners aged 3 to 6-years. This finding is plausible, because kindergarten teachers being overweight themselves have been shown to be less able to identify overweight in kindergartners [18], and it seems reasonable to assume that misclassification of overweight in children probably is another important risk factor of childhood overweight, especially in boys as demonstrated by the finding that parents misinterpret overweight more likely in boys.

## Conclusions

Although quite a number of studies have already identified risk factors of early life associated with overweight in children [12, 13], no study focused on child-caregivers and their potential association on children’s weight status so far. Here we show for the first time that kindergarten teachers’ weight status and habitual physical activity score are independent risk factors for childhood overweight. This association was robust against adjustment for the association of all of the other factors including the major parental factors that are known to be associated with overweight and that were also confirmed to be significantly associated in our study cohort. Psychosocial factors of parents associated with childhood overweight are likely to be biased by genetic factors and adjustment for this association cannot be undertaken properly, since the genetic factors including the epigenetic dimension and the gene environment interaction cannot yet be assessed properly. Our results here stress the relative importance of non-parental psychosocial factors for the development of childhood overweight and they may prompt a careful reconsideration of the dimension that genetic associations may have on childhood overweight.

Our results may provide a new direction for pediatric obesity research and ongoing public health approaches. Yet, it is already agreed that tailored prevention-interventions should start as early as possible and for practical reasons around the time of kindergarten entry [62]. This will hopefully counteract the rapid increase of childhood overweight that has recently been shown to focus around the general life-time event of school entry in Germany [10]. Although kindergarten teachers’ awareness of overweight-related health risks has been shown to be significantly higher compared to the awareness of the respective parents [18], there seems to be an urgent need of restructuring the kindergarten teacher training programs. In addition, to improving health education a call for personal lifestyle counseling for improving health related behavior in kindergarten teachers is now corroborated by both, the present weight and physical activity status of kindergarten teachers, as well as by the associated weight status of their kindergartners.

## Abbreviations

AGA: Appropriate for gestational age
ANOVA: Analysis of variance
HPAQ: Habitual physical activity questionnaire
BMI: Body mass index
CDC: Center for Disease Control and Prevention
CHSM: Children Health Study of Mainz
CI: Confidence intervals
CVD: Cardiovascular disease
DEGS1: German Health Interview and Examination Survey for Adults
DESTATIS: Federal Statistical Office
DVD: Digital versatile discs
h: hours
HPA: Habitual physical activity
IOTF: International Obesity Task Force
KiGGS: German Health and Examination Survey of Children and Adolescents
KTHS: Kindergarten Teacher Health Study
LGA: Large for gestational age
LSI: Life situation index
MVPA: moderate-to-vigorous physical activity
ORs: Odds ratios
PC: Personal computer
SD: Standard deviation
SDS: Standard deviation scores
SEP: Socio-economic position
SGA: Small for gestational age
TV: Television
WC: Waist circumference
WHO: World Health Organization.
